# Aggressive mimicry in a coral reef fish: The prey's view

**DOI:** 10.1002/ece3.6883

**Published:** 2020-10-20

**Authors:** Michele E. R. Pierotti, Anna Wandycz, Pawel Wandycz, Anja Rebelein, Vitor H. Corredor, Juliana H. Tashiro, Armando Castillo, William T. Wcislo, W. Owen McMillan, Ellis R. Loew

**Affiliations:** ^1^ Smithsonian Tropical Research Institute Balboa Panama; ^2^ Department of Anatomy, Institute of Zoology Jagiellonian University Krakow Poland; ^3^ Faculty of Geology, Geophysics and Environment Protection AGH University of Science and Technology Krakow Poland; ^4^ Thunen Institute Braunschweig Germany; ^5^ Department of Experimental Psychology, Psychology Institute University of São Paulo São Paulo Brazil; ^6^ Department of Biomedical Sciences Cornell University Ithaca NY USA

**Keywords:** communication, coral reef fish, mimicry, predation, sensory biology, visual signals

## Abstract

Since all forms of mimicry are based on perceptual deception, the sensory ecology of the intended receiver is of paramount importance to test the necessary precondition for mimicry to occur, that is, model‐mimic misidentification, and to gain insight in the origin and evolutionary trajectory of the signals. Here we test the potential for aggressive mimicry by a group of coral reef fishes, the color polymorphic *Hypoplectrus* hamlets, from the point of view of their most common prey, small epibenthic gobies and mysid shrimp. We build visual models based on the visual pigments and spatial resolution of the prey, the underwater light spectrum and color reflectances of putative models and their hamlet mimics. Our results are consistent with one mimic‐model relationship between the butter hamlet *H. unicolor* and its model the butterflyfish *Chaetodon capistratus* but do not support a second proposed mimic‐model pair between the black hamlet *H. nigricans* and the dusky damselfish *Stegastes adustus*. We discuss our results in the context of color morphs divergence in the *Hypoplectrus* species radiation and suggest that aggressive mimicry in *H. unicolor* might have originated in the context of protective (Batesian) mimicry by the hamlet from its fish predators rather than aggressive mimicry driven by its prey.

## Graphical abstract

Hamlets are colour polymorphic reef fishes believed to be diverging by aggressive mimicking of one of a number of unrelated non‐predatory fishes. We study the visual system of the intended receiver of hamlets' mimicry, their prey, and test whether model‐mimic mis‐identification by the prey is likely to occur. We find that only one hamlet is likely to be an efficient mimic in the eyes of their prey but we observe that the mimic colour morph might in fact have evolved by Batesian mimicry first and only subsequently recruited for aggressive mimicry.

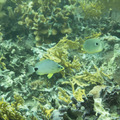

## 1 INTRODUCTION

Despite 150 years of research since Bates' (1862) and Wallace (1869)’s original insights, the unequivocal identification of new cases of mimicry, their evolutionary dynamics and the very definition and boundaries of the concept of mimicry are still challenging and hotly debated issues among evolutionary biologists (Dalziell & Welbergen, 2016; Moynihan, 1981; Rainey & Grether, 2007; Ruxton et al., 2004; Vane‐Wright, 1980; Wickler, 2013). A key realization that has emerged from the ongoing debate is that the perceptual system of the signal receiver must be put at the center of any analysis on the origins and maintenance of a mimicry system (Cuthill & Bennett, 1993; Dittrich et al., 1993). Indeed, testing hypotheses of mimic‐model relationships can be misleading without an appropriate eye‐of‐the‐beholder approach (Dittrich et al., 1993). This is because the evolution of a mimic signal is shaped not by similarity with the model but by the receiver's percepts of both the signals from model and mimic (Dalziell & Welbergen, 2016; de Jager & Anderson, 2019). Reducing the difference between those percepts below the receiver's threshold for detecting a just noticeable difference (sensu Fechner, 1860) will ensure effective mimicry.

It follows that high fidelity might not be the most important requirement for efficient mimicry. Cognitive processes such as generalization (Darst & Cummings, 2006; Ham et al., 2006), categorization (Chittka & Osorio, 2007) and overshadowing (Mackintosh, 1976), by which a conflict in the perception of multiple cues leads to only a subset of characteristics of the signal being considered at the expense of others, are likely to affect mimetic accuracy. By acting on the receiver percept of the mimic phenotype and not on the phenotype itself, selection will frequently affect only a limited subset of traits in the mimic, those most salient for the sensory system of the intended receiver. Indeed, selection might even drive the evolution of “imperfect” mimics with higher mimicry performance than high fidelity mimics, for example by enhancing salient signals beyond the value characteristic of the model, to increase effectiveness of recognition, memorization or more effective receiver manipulation (Kilner et al., 1999). In addition, a receiver percept results from alterations of the signal as it travels through the medium (e.g., air, water) from the model or mimic to the receiver's sensory system. Characteristics of the medium (e.g., its general physical properties or those of the background against which the model/mimic are seen or heard) might enhance or attenuate certain components of the signal making perfect imitation of the model unnecessary. In conclusion, evidence in support of a particular putative mimic‐model relationship needs to be grounded in an understanding of the receiver's perceptual system and its sensory environment.

An intriguing putative case of mimicry is represented by the *Hypoplectrus* hamlet complex, a group of coral reef fish with strikingly distinct color patterns. Despite assortative mating by color morph, which led various authors to recognize them as separate species, hamlets exhibit otherwise little morphological and genetic differentiation between morphs at any one locality (Aguilar‐Perera & González‐Salas, 2010; Graves & Rosenblatt, 1980; McCartney et al., 2003; Puebla et al., 2008, 2014; Whiteman & Gage, 2007). Indeed, in a recent genome‐wide analysis, hamlet species only consistently differed from each other at genomic regions that contained loci implicated in the production or perception of color pattern (Hench et al., 2019). Various authors have suggested that the hamlets' exceptional color diversity might be the result of aggressive mimicry of a number of non‐predator model species by different hamlet morphs (Domeier, 1994; Fischer, 1980; Holt et al., 2008; Puebla et al., 2018; Randall & Randall, 1960; Thresher, 1978; Whiteman et al., 2007). Here, the mimic species takes the appearance of a non‐predatory species in order to get close to a potential prey, small fish and epibenthic invertebrates, without eliciting an escape reaction.

For those hamlet morphs considered mimics, one or more candidate models have been proposed, typically co‐occurring herbivore, corallivore or spongivore fish species, harmless to hamlets’ prey and exhibiting various degrees of resemblance, as judged by a human viewer, to the corresponding hamlet morph (Domeier, 1994; Fischer, 1980; Puebla et al., 2007; Randall & Randall, 1960; Thresher, 1978). However, the plausibility of aggressive mimicry in hamlets rests only on these apparent color pattern similarities. A notable exception is the work of Puebla and coworkers on a butter hamlet *H. unicolor* population in Panama (Puebla et al., 2007, 2018). The authors showed that the proportion of butter hamlet strikes toward their prey was significantly higher when associating with their putative model, the four‐eye butterflyfish *Chaetodon capistratus*, than when striking alone, suggesting a possible fitness advantage consistent with aggressive mimicry in the butter hamlet.

Despite frequent reference to aggressive mimicry as an evolutionary engine of hamlet diversification (Domeier, 1994; Fischer, 1980; Puebla et al., 2007; Thresher, 1978), we still lack a basic understanding of hamlet preys’ visual abilities and their potential for effective discrimination of predatory hamlet color morphs from harmless (putative) models. Here we consider three widely distributed hamlet species, the putative mimics butter hamlet (*H. unicolor*) and black hamlet (*H. nigricans*), and the non‐mimic barred hamlet (*H. puella*). We examine the visual system of two of their most common prey, namely an epibenthic coral reef fish and an open‐water mysid shrimp, both in terms of color vision and visual acuity. Using spectral reflectance measurements of equivalent patches on each hamlet morph and their putative models and modeling of preys’ visual sensitivity and acuity, we gain insight into the potential for deception of each mimic hamlet morph through the eyes of their prey.

## 2 MATERIALS AND METHODS

### 2.1 Study site and species

Field work was conducted in the Bocas del Toro Archipelago, Panama, on the same reef complex (Punta Caracol; GPS 9°21'38.449'' N, 82°16'40.803'' W), where the association between *H. unicolor* hamlets and the butterflyfish *C. capistratus* had been previously observed by Puebla et al. (2007). Fish were collected while SCUBA diving by hook‐and‐line or with hand nets at depths of between 3−8 m and then kept briefly in 80 cm × 80 cm × 50 cm outdoor aquaria with running seawater, before data collection.

We considered a non‐mimic hamlet, the barred *H. puella*, and two model‐mimic putative pairs: (i) the butter hamlet (*H. unicolor*) and its model, the four‐eye butterflyfish (*Chaetodon capistratus*); (ii) the black hamlet (*H. nigricans*) and its model the dusky damselfish (*Stegastes adustus*). In addition, we collected the two most common hamlet prey encountered in hamlet stomach contents, in the Bocas del Toro populations (Puebla et al., 2007), the masked goby (*Coryphopterus personatus*) and a mysid shrimp (*Mysidium columbiae*). *C. personatus* is a microbenthic goby occurring in large schools hovering above coral heads and feeding on plankton (Böhlke & Robins, 1962). *Mysidium columbiae* are among the most abundant swarming planktonic crustaceans on shallow coral reefs in the Gulf of Mexico and the Caribbean, generally found in patchy aggregations over corals or among mangrove roots (Wittmann & Wirtz, 2019).

### 2.2 Spectral measurements

#### 2.2.1 Underwater spectral irradiances

We characterized the underwater photic environment on the Punta Caracol shallow coral reefs (Bocas del Toro, Panama) where the hamlet morphs, their models and their prey were collected by measuring spectral irradiances just under the surface and at 2.5 m, 5.0 m and 7.5 m depth above hamlet territories, from downwelling, upwelling and sidewelling underwater light (detailed methods in Appendix 1).

#### 2.2.2 Fish body reflectance

A number of equivalent areas on the fish body showing clear differences in coloration between two or more species were identified and used to build a set of 7 “homologous” landmarks, shared by all hamlets and their putative models. Reflectance measurements were taken from these landmarks across species, with a 200 µm UV/vis bifurcated fiber optic cable connected to an Ocean Optics USB2000 spectrometer and an Ocean Optics PX‐2 pulsed xenon light source. Individuals were placed over a damp cloth and their skin maintained wet, following the guidelines of Marshall (2000). Reflectance records, obtained keeping both ends of the bifurcated fiber at approximately 45 degrees above the color patches, were calibrated against a Spectralon (Labsphere, North Sutton, NH) white standard.

### 2.3 Receivers’ visual system

#### 2.3.1 Masked goby

We characterized the spectral sensitivity of the masked goby visual pigments by microspectrophotometry, as in Loew (1994) (detailed in Appendix 1). Lens transmission was measured directing light from the pulsed xenon light source through the lens mounted on a pinhole into a UV/vis fiber optic cable connected to the Ocean Optics USB2000 spectrometer. Lens transmission was expressed in terms of the 50% cutoff wavelength (*T*_50_) calculated from transmission spectra normalized to their maximum transmission between 300–700 nm.

#### 2.3.2 Mysid shrimp

Opossum shrimp (Crustacea: Mysida) have superposition eyes (Hallberg, 1977). Visual sensitivity has been studied in depth in the genus *Mysis*, with evidence for a single visual pigment, with peak of spectral absorbance (*λ*_max_) positioned in the waveband 520–525 nm in marine populations (Audzijonyte et al., 2012; Jokela‐Määttä et al., 2005). In the absence of available data on visual sensitivities in *Mysidium*, we chose a value of 520 nm, consistent with values in the related *Mysis*.

### 2.4 Color and luminance discrimination

Vorobyev and Osorio’s (1998) color discrimination model was used to derive chromatic distances between corresponding color patches in hamlets, their models and natural backgrounds as perceived by a masked goby and by a mysid shrimp (visual modeling detailed in Appendix 1). The model assumes that the limits to color discrimination are set by noise arising at the photoreceptor level and color distances are expressed in multiples of the theoretical detection threshold, the just noticeable difference (JND). We tested whether perceptual distances in color (dS) and luminance (dL) of corresponding patches on hamlets and their putative models were statistically different with a PERMANOVA approach, using color and luminance distances in just noticeable difference units (JNDs), with the *adonis* function in the R package *vegan* (Oksanen et al., 2007). Prior to this, we tested the assumption of homogeneity of multivariate dispersions for each patch across species using a multivariate extension of Levene's test for homogeneity, with the “*betadisper*” function in *vegan*, followed by Tukey's HSD test to inspect differences between the multivariate dispersions (Anderson, 2006; Seber, 1984). The distance‐based PERMANOVA analysis was used to generate a pseudo‐F statistics from the ratio of among/within distances between groups, and to obtain a null distribution by randomizing distances between observations (Anderson, 2005). We used 999 permutations to test for significant deviation from the null distribution and used the *R* ^2^ as an estimate of effect size. Post hoc tests were performed with the *pairwiseAdonis* function (Martinez Arbizu, 2019), returning adjusted *p*‐values on the pairwise comparisons.

We then assessed whether the effect size of the above differences between model and mimic was sufficiently large for the visual system of the receiver (i.e., the prey: masked goby, mysid shrimp) to perceive them, using a bootstrap procedure (Maia & White, 2018) as implemented in *pavo*. For each bootstrap run, Maia and White (2018) approach samples points (patches) from each group (species) of the same size of the original group and with replacement and then estimates the distance in color space, based on noise‐corrected quantum catches, between the geometric means of the bootstrap resample. Bootstrapping allows to generate a distribution of these means and a confidence interval to be calculated. Confidence intervals above the perceptual threshold are considered likely discriminable by the viewer. All analyses were run separately using the mysid shrimp and the masked goby visual systems, the latter under both a dichromatic and a trichromatic scenario.

### 2.5 A prey's view of natural scenes

We wished to gain insight into the spatial information available to masked gobies and mysid shrimp, when attempting the discrimination of a hamlet from its model in a natural scene. We applied the method of Caves and Johnsen (2018), implemented in the R package *AcuityView* (R Development Core Team, 2016) which uses a Fourier transform approach to convert an image from spatial into frequency domain, then multiply it pixel by pixel by a modulation transfer function (MTF). This method uses Snyder's (1977) MTF, which is a function of the minimum resolvable angle of the viewer. The result is an image that is devoid of all the frequencies above a threshold corresponding to the viewer's acuity. After inverse Fourier transform to spatial domain, the image retains the level of detail that lies above the contrast threshold dictated by the viewer's acuity and therefore provides us with insight into the information content available to the prey viewing a scene including a predator, the hamlet, or a harmless “passer‐by,” the model species (image collection and processing detailed in Appendix 1).

Underwater observations of feeding hamlets in Bocas del Toro populations (*pers. obs*., 2015–2017) showed that they can rush from a distance of more than 4ft toward a dense cloud of mysids or masked gobies, stopping for about 1–2 s at a distance of less than 50 cm (i.e., about five body lengths) away from the prey, for precision aiming at a single individual. They then strike horizontally when preying on mysids and from above at about 45° when striking at masked gobies. Given this pattern of predatory behavior, we considered that a mysid or a masked goby would have a reasonable chance of evading a predatory strike if it recognized an approaching predator and initiated escape at a distance over three hamlet body lengths away, that is, about 25 cm distance. We used these two values, 50 cm and 25 cm as the relevant viewing distances d, in the image analysis. The angle *α* subtending the scene is then *α* = 2arctg(actual width/2d).

#### 2.5.1 Masked goby acuity

We assessed the acuity of the masked goby *C. personatus* in terms of its optical anatomy, based on ganglion cell densities (Coimbra et al., 2012; Collin & Pettigrew, 1988a, 1988b). Therefore, we consider these estimates as representing an upper limit to the masked goby acuity. To do this, we analyzed the population of ganglion cell layer (GCL) cells and estimated the upper limits of spatial resolving power from two retinas from two *C. personatus* individuals. (see Appendix 1 for protocol).

#### 2.5.2 Mysidium shrimp acuity

Behavioral estimates of visual acuity are available for the mysid shrimp *Mysidium columbiae*, based on optomotor response experiments (Buskey, 2000). Under conditions of optimal illumination, individuals placed in an optokinetic drum, followed vertical black and white stripes of varying width consistently resolving differences down to 6 mm from a minimum distance of 15 mm. Given the known relationship between subtended angle *α*, reactive distance d and stripe width *w*: *α* = 2arctg(0.5*w*/*d*), this corresponds to a subtending arc of 7.66 degrees, the value of behavioral acuity we used in the spatial patterns analyses.

## 3 RESULTS

### 3.1 Spectral measurements

#### 3.1.1 Underwater spectral irradiances

**FIGURE 1:**
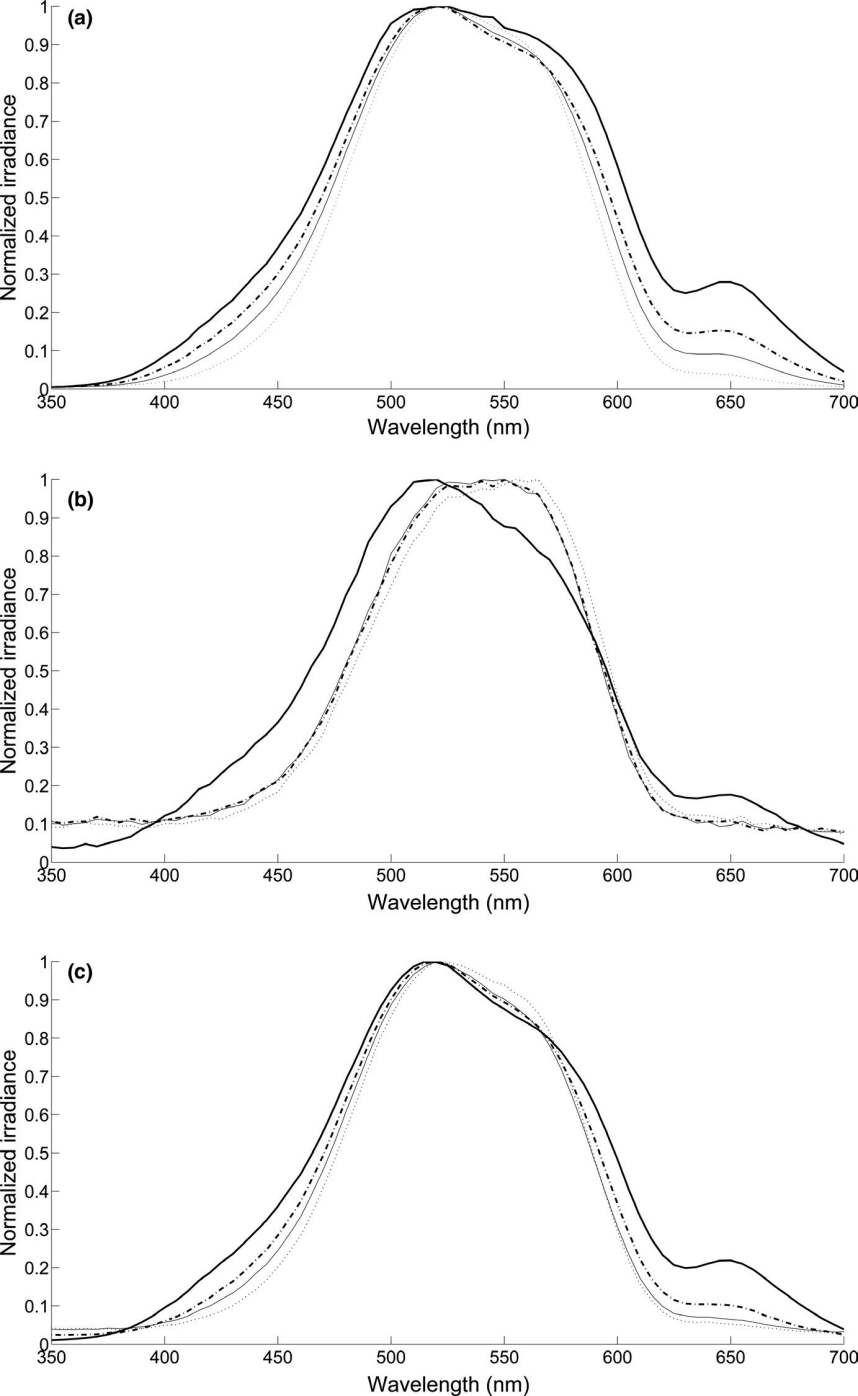
Downwelling (*top*), upwelling (*center*), and average sidewelling (*bottom*) irradiances, measured at Punta Caracol, Bocas Del Toro, Panama, on a vertical depth profile, just under the surface (**—**), 2.5 m (—), 5.0 m (‐‐‐) and 7.5 m (⋯)

**FIGURE A1:**
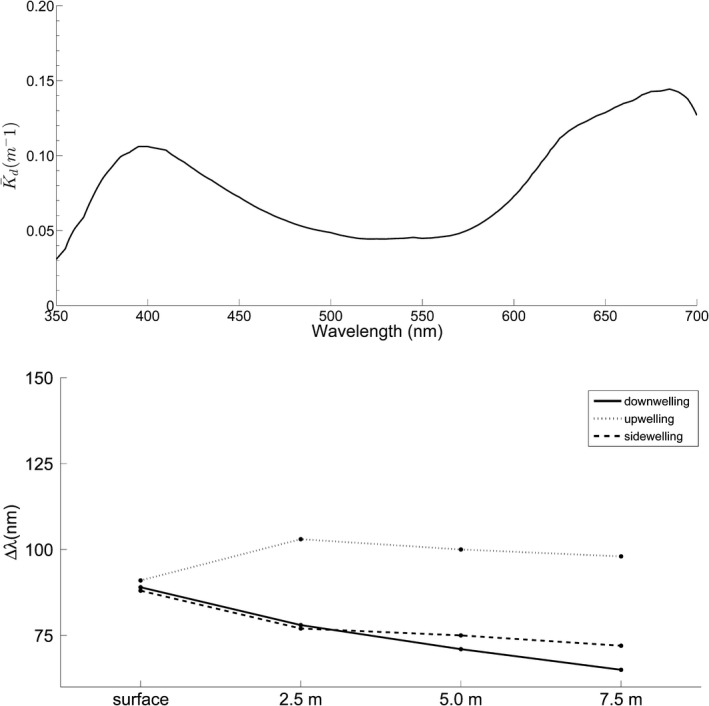
*Top*: Average diffuse attenuation coefficient ${\bar{K}}_{d}\left(z,\lambda \right)$ (over the 0 ÷ 7.5 m depth interval, above hamlet territories, at Punta Caracol, Bocas del Toro, Panama; *Bottom*: Spectrum bandwidth (Δ*λ*) of down‐, up‐ and averaged sidewelling irradiance, from below surface to close to bottom of reef (7.5 m)

Downwelling spectral irradiance over our study site had typical characteristics of tropical reef waters (Figure 1) with similar *λ*P_50_ (the wavelength that halves the total number of photons, Munz & McFarland, 1973), across depths (under the surface: 516 nm; 2.5 m: 516 nm; 5.0 m: 512 nm; 7.5 m: 520 nm) and values very close to those measured by McFarland and Munz (1975) on a Pacific atoll (*λ*P_50_ = 518 nm at 5 m depth) (Figure 1 main text and Figure A1 in Appendix 1).

Upwelling irradiance at maximum depth, closer to the bottom of this shallow reef system, was shifted to longer wavelength (Figure 1) as a result of the mixed composition of the bottom, consisting of coral outcrops interspersed with yellow sand patches. As the distance from the bottom increased, the upwelling spectrum shifts to shorter wavelengths and to a spectral profile similar to the downwelling and sidewelling spectra (see also Appendix 1).

#### 3.1.2 Fish body reflectances

**FIGURE 2:**
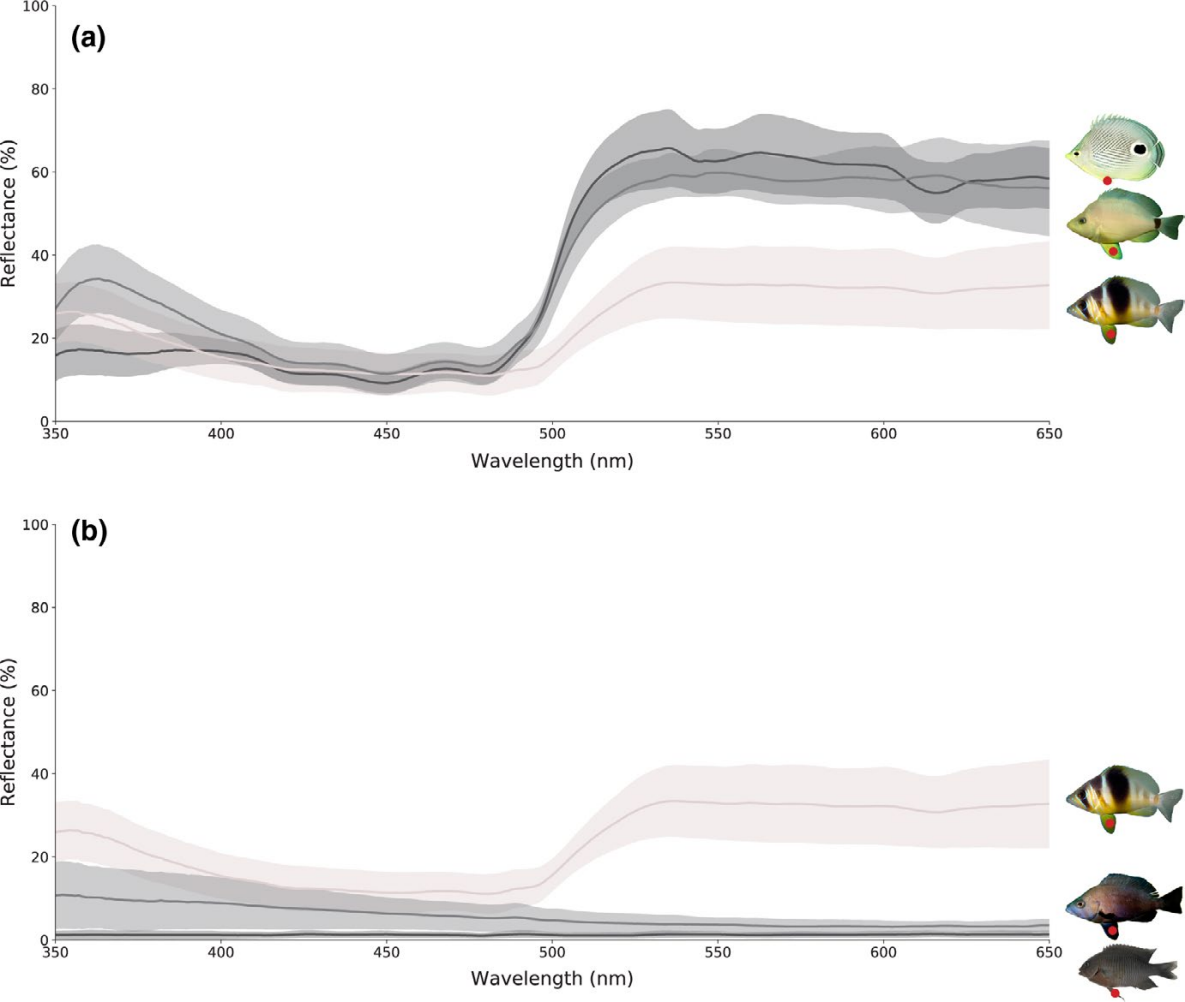
Mean spectral reflectance and standard deviation of body patch #5 (center of left pelvic fin, indicated here as a red spot on the images) across species. *Top*: from top, four‐eye butterflyfish *C. capistratus* (putative model 1), butter hamlet *H. unicolor* (putative mimic 1) and the non‐mimic barred hamlet *H. puella*. *Bottom*: from top, the non‐mimic barred hamlet *H. puella* and the black hamlet *H. nigricans* (putative mimic 2), and bottom, the dusky damselfish *S. adustus* (putative model 2)

Spectral reflectance measurements were collected from 7 homologous landmarks on the body of 42 individuals, namely *H. puella* (*n* = 10), *H. nigricans* (*n* = 9), *H. unicolor* (*n* = 7), *C. capistratus* (*n* = 8) and *S. adustus* (*n* = 8). Mean reflectance spectrum and standard deviation for a representative spot (pelvic fin) are shown in Figure 2.

### 3.2 Receivers visual system

#### 3.2.1 Masked goby visual sensitivity

**FIGURE 3:**
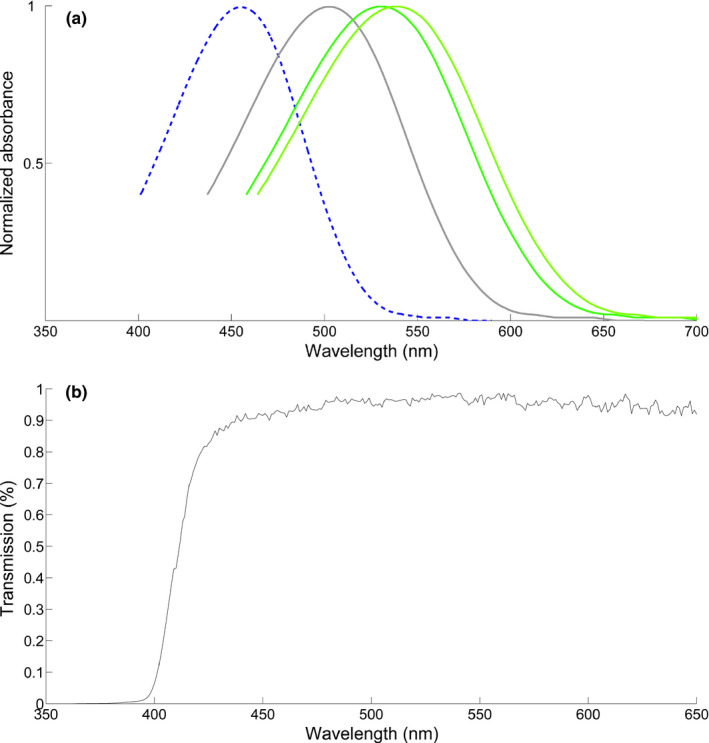
*Top*: Absorbance templates (from Govardovskii et al., 2000; Lipetz & Cronin, 1988), representing the rod (501 nm; in gray) and cone visual pigments (531 nm, 539 nm; in green) found in the retina of the goby *Coryphopterus personatus*; an additional blue cone pigment (455 nm; in blue) was added, in an alternative modeling scenario (see main text). *Bottom*: Lens transmission spectrum of the masked goby *C. personatus*. The *T*_50_ is located at 410 ÷ 411 nm

**FIGURE 4:**
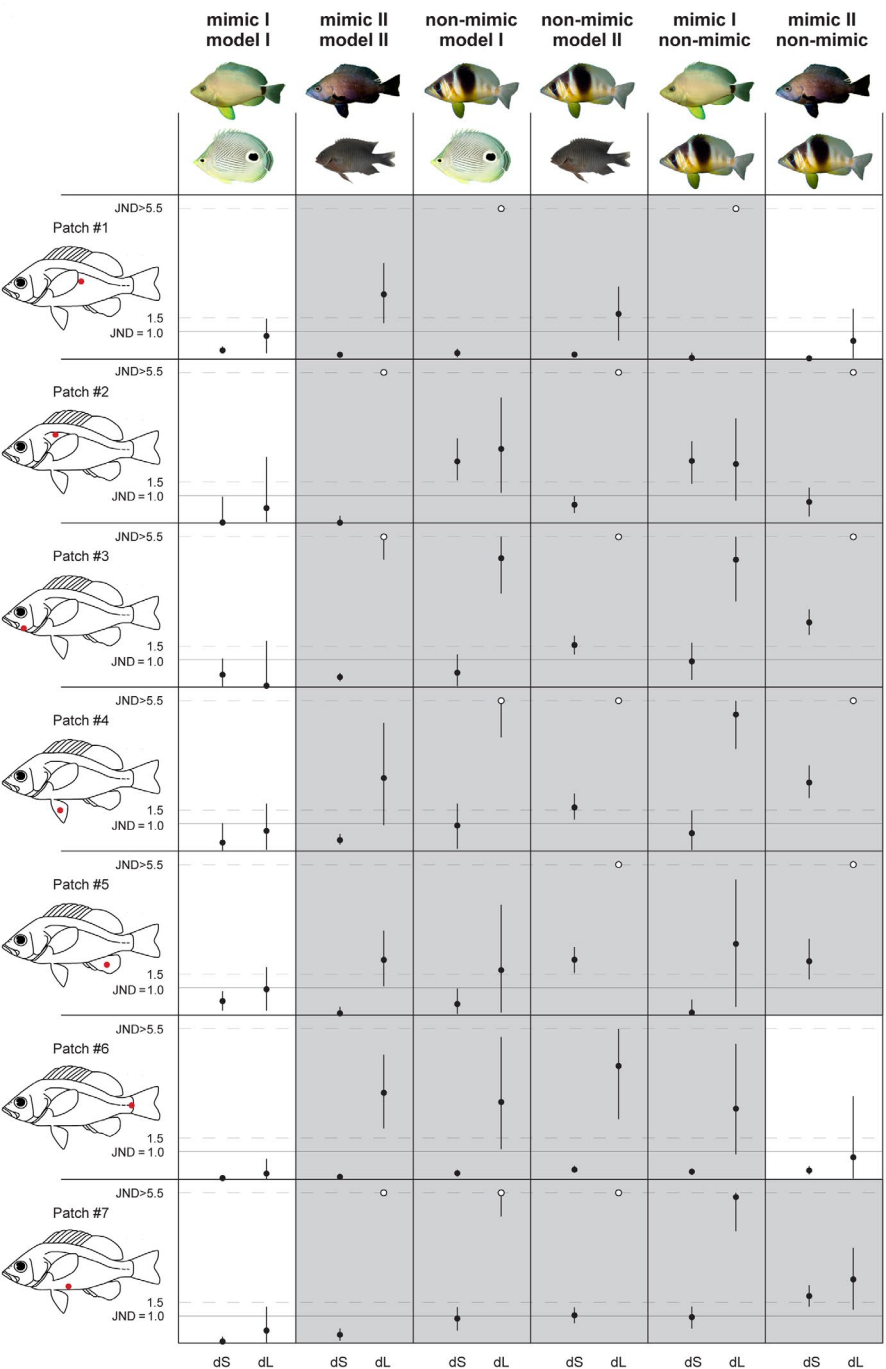
Color distances dS (on the left, in each box) and achromatic distances dL (on the right, in each box) and their 95% bootstrap confidence limits, for each species pair viewed by the prey, a masked goby visual system with 455 nm, 531 nm, 539 nm cone pigments, at a depth of 5 m. A continuous line, corresponding to dS (or dL) = 1, marks the perceptual threshold, below which a particular patch (#1–7, indicated in the fish silhouette on the left) is likely indistinguishable by the viewer. When distances dS, dL are larger than 5.5 units of JND, they are presented with an open dot at the top end of their respective box. Discriminable patches (either because dS or dL > 1, or both) are represented by gray‐shaded boxes

**FIGURE A2:**
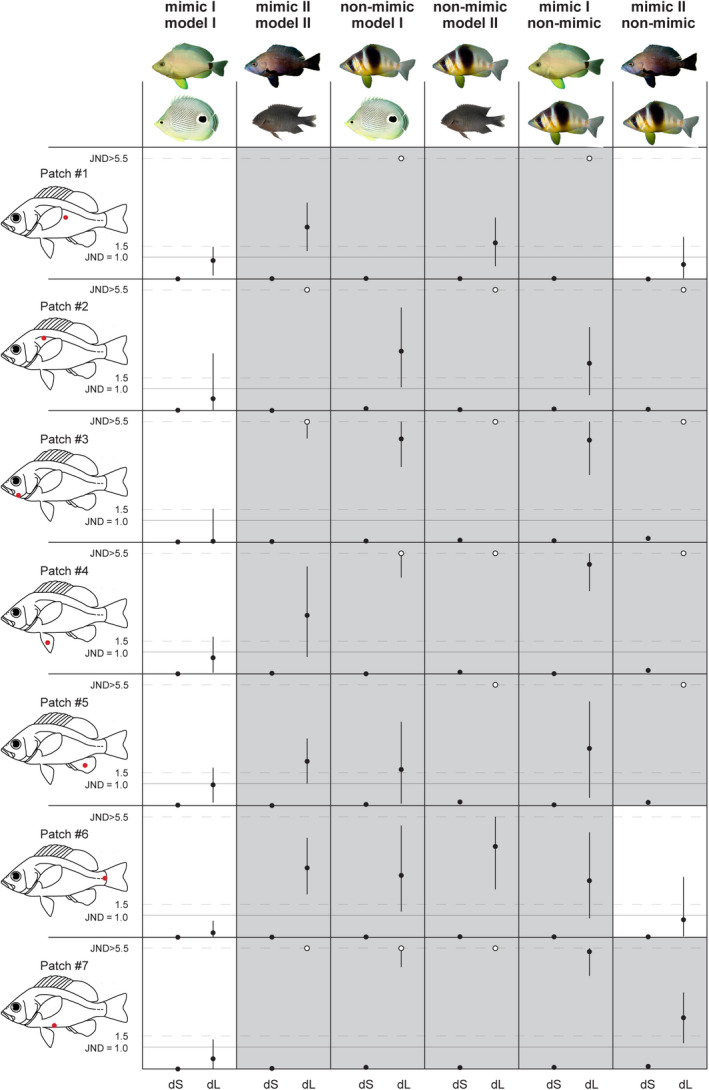
Color distances dS (on the left, in each box) and achromatic distances dL (on the right, in each box) and their bootstrap confidence limits, for each species pair viewed by the prey, a masked goby with a 531/539 nm set of cone pigments, at a depth of 5 m. A value of dS (or dL) = 1 marks the theoretical perceptual threshold for each patch (#1–7). Figure conventions as in Figure 4, main text

We collected spectral sensitivity of both rods and cones from the retinas of *n* = 4 *C. personatus* individuals. The retina of masked gobies appeared rod‐dominated (rods mean and *SD*: *λ*_max_ = 500.2 ± 1.48 nm, range 497 ÷ 503, *n* = 17). Double cones showed either the same visual pigment at 539 nm (*λ*_max_ = 538.8 ± 1.28; range 537 ÷ 541; *n* = 13) or one member with *λ*_max_ = 539 nm and the other with *λ*_max_ = 531 nm (*λ*_max_ = 530.9 ± 1.51; range 527 ÷ 533; *n* = 21). We did not find any evidence for short‐wavelength cones. The very small distance between the two green‐sensitive cone *λ*_max_ (531 nm, 539 nm) is unlikely to provide the masked goby with true color vision (see also the results of the visual models with dichromatic and trichromatic vision, Figure 4 and Figure A2) but might confer the fish broader sensitivity in that particular region of the light spectrum. Overall, these results are in line with previously reported spectral sensitivities from other tropical gobies (Table A1). In particular, the absence of a short‐wavelength cone in *C. personatus* is a condition shared with the only other coral reef goby for which MSP data are available. However, we cannot exclude that small blue or violet cones might be present in very low frequencies in the masked goby retina, given the sampling design typical of microspectrophotometry. For this reason (see below), we considered both a visual model that includes the cone repertoire found by our MSP study and an additional one that incorporates a third short‐wavelength cone, positioned in the region of sensitivity characteristic of other gobies (*λ*_max_ ≈ 455 nm), that would potentially confer trichromatic color vision to the goby (Figure 3 *top*, main text; Table A1, Appendix 1).

#### 3.2.2 Masked goby lens transmittance

We examined the eye lenses of *n* = 3 individuals. The wavelength at which 50% of the maximal transmittance was reached (*T*_50_ = 410÷411 nm) and the shape of the transmittance curve suggest that there is little scope for UV signals reception in this goby species (Figure 3, *bottom*).

### 3.3 Color and luminance discrimination

#### 3.3.1 Masked goby

The PERMANOVAs of differences in dS and dL between corresponding patches across species, calculated at different depths, and with either two (531 nm, 539 nm) or three (455 nm, 531 nm, 539 nm) cone classes, were all significant (*p* < .001; Table S1‐Dryad Archive). Post hoc tests on chromatic distances dS showed that for almost all species pair contrasts, at least three or more patches on the body were significantly different between species. The exceptions were the two putative model‐mimic pairs: the butter hamlet *H. unicolor* and its model, the butterflyfish *C. capistratus*, were not significantly distinguishable in color at any of the measured patches, when the viewer had the 531/539 nm cone set and distinguishable by one patch (#1) only, when the viewer was provided with a 455/531/539 nm cone set, and this irrespective of depth. In the black hamlet *H. nigricans* and its putative model, the damselfish *S. adustus*, two patches (#3, #7) were significantly different in color between species when seen by a 531/539 nm viewer and three patches (#1, #3, #7) when seen by a 455/531/539 nm viewer, although not at all three depths (Table S1‐Dryad Archive). Post hoc tests on achromatic distances dL reveal that at least four and up to all seven patches were significantly different (*p* < .05) across species at any depth, with the only exception of the model‐mimic pair butter hamlet *H. unicolor* and butterflyfish *C. capistratus* for which all seven patches were indistinguishable (*p* > .21) between species, irrespective of depth and cone set of the goby.

**FIGURE 5:**
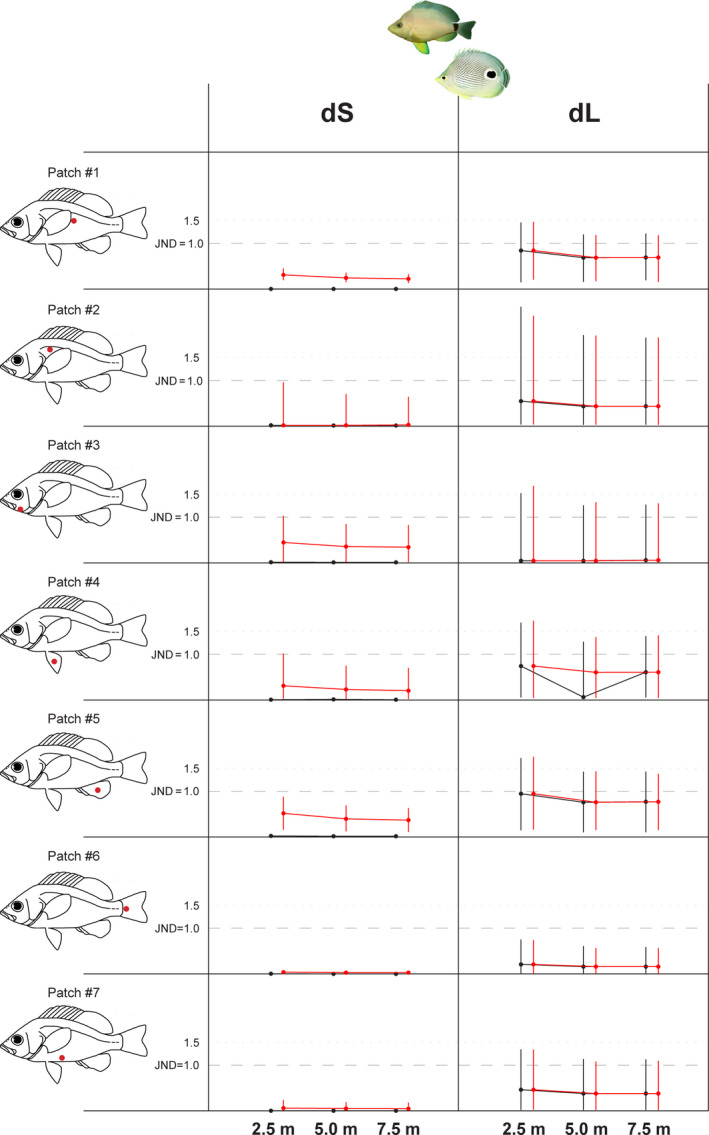
Chromatic (dS) and achromatic distances (dL) and their 95% bootstrap confidence limits, for the mimic‐model pair *H. unicolor* and *C. capistratus*, at different depths (2.5 m, 5.0 m, 7.5 m), viewed by the prey, a masked goby with a trichromatic 455 nm, 531 nm, 539 nm visual system (red lines) or a dichromatic 531 nm, 539 nm visual system (black lines)

Whether the significant differences in color and luminance found between species are of a magnitude detectable by the viewer (perceptual effect size) was tested by calculating distances between the geometric means of each species and generating confidence intervals by bootstrapping (Maia & White, 2018). We found that the only patch that was significantly different in the PERMANOVA analysis between the *H. unicolor–C. capistratus* mimic‐model pair, when the goby was provided with three visual pigments 455/531/539, is effectively indistinguishable by the goby (dS < 0.31) at all depths, as are all other patches (Figure 4), and this holds true irrespective of depth and cone pigment repertoire of the goby (Figure 5 and Figure A2, Table S2‐Dryad Archive). This result suggests that the *H. unicolor–C. capistratus* pair fulfils the requirement of a model‐mimic relationship, in terms of color differences since color distances between corresponding patches on model and mimic are well below the discrimination threshold of the signal receiver, the goby. In the other putative mimic‐model pair, *H. nigricans*–*S. adustus*, the various patches that we found significantly different in the PERMANOVA analysis, had color distances of magnitude below or at the discrimination threshold (Figure 4), and that holds true at all depths and cone set conditions (Table S2‐Dryad Archive), in line with a model‐mimic hypothesis. All other species pair contrasts were above threshold when modeled with a 455/531/539 cone set, while chromatic contrasts between the pair *S. adustus–C. capistratus* were below threshold when modeled with a 531/539 goby visual system (Table S2‐Dryad Archive).

The bootstrap analysis of achromatic contrasts revealed that for one species pair only, the putative mimic–model pair *H. unicolor–C. capistratus,* luminance distances were always below threshold (mean dL < 1 for all patches, trichromatic goby: Figures 4 and 5), consistent with a model‐mimic scenario. All other species contrasts contain more than one patch that can be discriminated between species by the goby based on achromatic distances, including the second putative pair, *H. nigricans–S. adustus* (Figure 4 and Figure A2, Table S3‐Dryad Archive).

#### 3.3.2 mysid shrimp

The PERMANOVAs of differences in dL between corresponding patches across species, modeled with a single visual pigment and calculated at different depths were all significant (*p* < .004). Post hoc tests showed that at least four and up to all seven patches were significantly different (*p* < .05) across species at any depth, with the single exception of the model‐mimic pair butter hamlet *H. unicolor* and butterflyfish *C. capistratus* for which all seven patches were indistinguishable (*p* > .23) between species, irrespective of depth (Table S3‐Dryad Archive).

**FIGURE A3:**
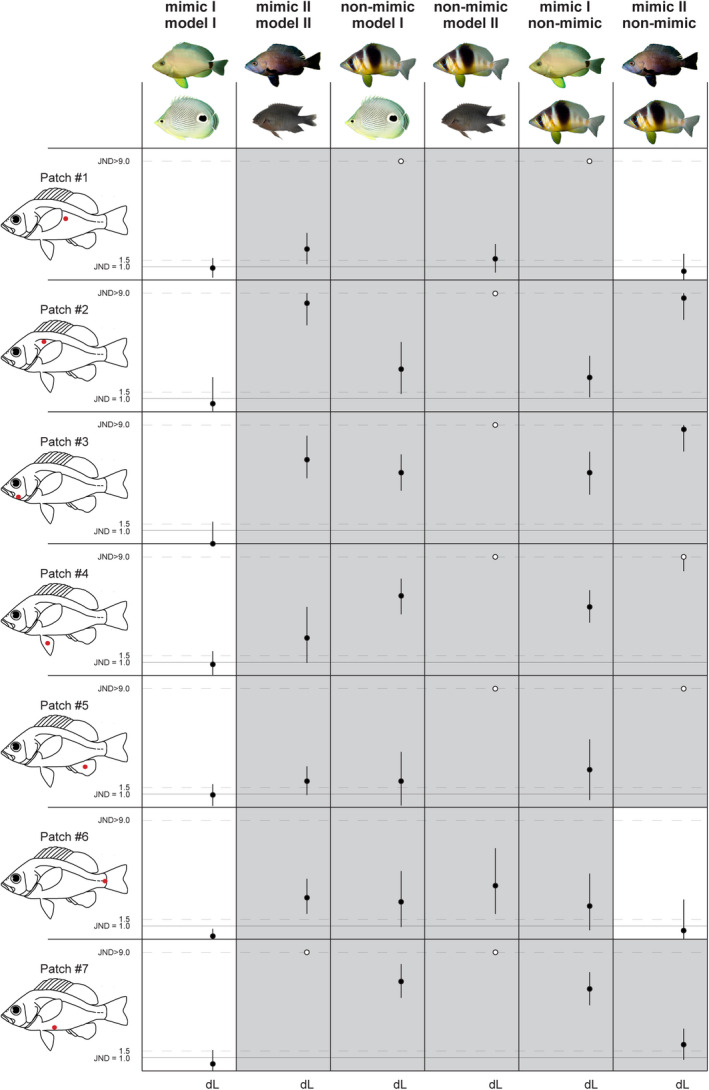
Achromatic contrasts dL across species, viewed by a *Mysidium columbiae* shrimp visual system with a single pigment of *λ*_max_ = 520, at a depth of 5 m. Conventions as in Figure 4, main text

The bootstraps of species groups showed that at least five patches were above detection threshold (dL > 1) in any two species contrast (Figure A3, Table S4‐Dryad Archive). The notable exception is the pair *H. unicolor*–*C. capistratus*, for which all spots were below theoretical detection threshold (dL < 1), suggesting that putative mimic (*H. unicolor*) and model (*C. capistratus*) are unlikely to be discriminable by the mysid shrimp based on achromatic contrast between corresponding patches. The achromatic differences in the second putative pair, *H. nigricans*–*S. adustus*, are all above threshold.

### 3.4 A prey's view of natural scenes

Visual acuity in the masked goby was estimated from the total number of cells in the ganglion cell layer of two retinal wholemounts from different *C. personatus* individuals. An average cell population of 179,769 ± 12,914 was obtained (Table 1), with high densities observed in the retinal periphery and peak density located in the ventral region (89,400 cells/mm^2^) while lowest densities were observed in the central retina (1,200 cells/mm^2^). The upper limit of the spatial resolving power estimated from lens radius and the maximum density of GCL cells was 2.356 ± 0.14 cycles/deg, corresponding to a minimum resolvable angle (MRA) *α* = 0.425 ± 0.02 degrees (Table 1). The number of sites counted for each retina, Scheaffer's coefficient of error (CE) and area of sampling fraction (asf) are described in Table 1. Shrinkage was below 5% and considered negligible (Coimbra et al., 2006). For the mysid shrimp, we used Buskey (2000)’s acuity estimate of *α* = 7.66 degrees (or 0.13 cycles/degree).

The underwater images taken at Punta Caracol and Punta Juan, in Bocas del Toro, Panama, were modified to account for the spatial resolution of *C. personatus* masked gobies and *Mysidium* shrimp. After processing, they provide a first approximation of scenes including models or putative aggressive mimics, as perceived by the prey, masked goby or mysid shrimp, given their visual acuity. The Fourier analysis of natural scenes suggests that *Mysidium columbiae* shrimp are only able to discern, even at relatively small distances (25 cm) bright moving versus dark moving objects in their field of view. While temporal resolution was not considered in this study, overall the visual system of this mysid shrimp would be unable to distinguish, at the distances considered, a harmless moving target from a predator, based on visual cues, with the exception, possibly, of information regarding the target's direction. The visual acuity of masked gobies *C. personatus*, in contrast, was sufficient to gather relevant information about other species’ general features at both distances, despite poor color vision. In particular the dark vertical bars of *H. puella* (and their absence in the mimic *H. unicolor*) and the eyespot in the posterior region of both model *C. capistratus* and mimic *H. unicolor* remain conspicuous features in an otherwise uncharacterized image.

**TABLE 1:** Stereological assessment of the population of cells in the retinal GCL of *Coryphopterus personatus* and the anatomical parameters used to estimate the upper limit of spatial resolution

Individual	Retinal area (mm^2^)	Sites counted	asf	Total number of GCL cells	CE	Mean density of GCL cells (cells/mm^2^)	Peak density of GCL cells	Lens diameter (mm)	Spatial resolving power in cycles/deg (MRA in degrees)
#1	5.3	190	0.09	170,638	0.046	32,196	84,000	0.83	2.456 (0.407)
#2	4.0	143	0.09	188,901	0.058	47,085	94,800	0.72	2.257 (0.443)
Mean ± *SD*	4.7 ± 0.9			179,769 ± 12,914		39,641 ± 10,529	89,400 ± 7,637		2.356 ± 0.14 (0.425 ± 0.02)

asf, area of sampling fraction; CE, Scheaffer’s coefficient of error; GCL, ganglion cell layer; MRA, minimum resolvable angle.

## 4 DISCUSSION

The extraordinary variation in color patterns between *Hypoplectrus* hamlets has long been attributed to aggressive mimicry (Domeier, 1994; Fischer, 1980; Puebla et al., 2007; Randall & Randall, 1960; Thresher, 1978). However, apart from such comparisons frequently based on color flash‐enriched photographs, no attempt has been made to examine the resemblance of hamlets to the proposed models from the meaningful perspective of the intended signal receiver under natural light conditions. In a case of aggressive mimicry such receiver is a hamlet prey. Here we examined two putative mimicry pairs in the *Hypoplectrus* hamlet complex as well as a non‐mimic hamlet, from the point of view of the visual system of two ecologically and taxonomically distinct prey species, the masked goby *C. personatus*, and the planktonic mysid shrimp, *M. columbiae*. We evaluated the prey's discriminating ability in terms of their visual acuity and their perception of hue and luminance and considered the effects of depth on the efficacy of mimicry. We found that one putative model‐mimic pair, the butter hamlet and the four‐eye butterflyfish, are the only pairwise species comparison well below the threshold of discriminability in the eyes of the prey. This, together with behavioral evidence for a fitness advantage gained by this hamlet morph when associating with its model butterflyfish and the presence of mimicking behavior (Picq et al., 2019; Puebla et al., 2007), constitutes strong support for an aggressive mimicry scenario in this pair. In a second putative model‐mimic relationship (*H. nigricans* black hamlet–S*. adustus* dusky damselfish), on the other hand, we observed large differences in perceived luminance for some patches (as expressed by double‐digit multiples of a just noticeable difference). This suggests that black hamlet and dusky damselfish might be distinguishable by hamlet prey on the basis of those differences, casting some doubts on their model‐mimic relationship.

Depth had very limited influence on visual thresholds, not surprisingly given the narrow depth range in which the majority of hamlet territories were located in our study population. In both chromatic models, the ability of the masked goby to discriminate the predator butter hamlet (*H. unicolor*) from its putative model, the four‐eye butterflyfish (*C. capistratus*) was well below the theoretical noise threshold both in terms of color and luminance, and often close or equal to zero. While caution is needed in the interpretation of perceptual thresholds in Vorobyev and Osorio (1998)’s receptor noise‐limited model, in particular given the absence of direct measurements of photoreceptor noise in the masked goby, modeling suggests that the prey would not be able to separate model from mimic based on differences in hue or brightness, regardless of depth.

**FIGURE 6:**
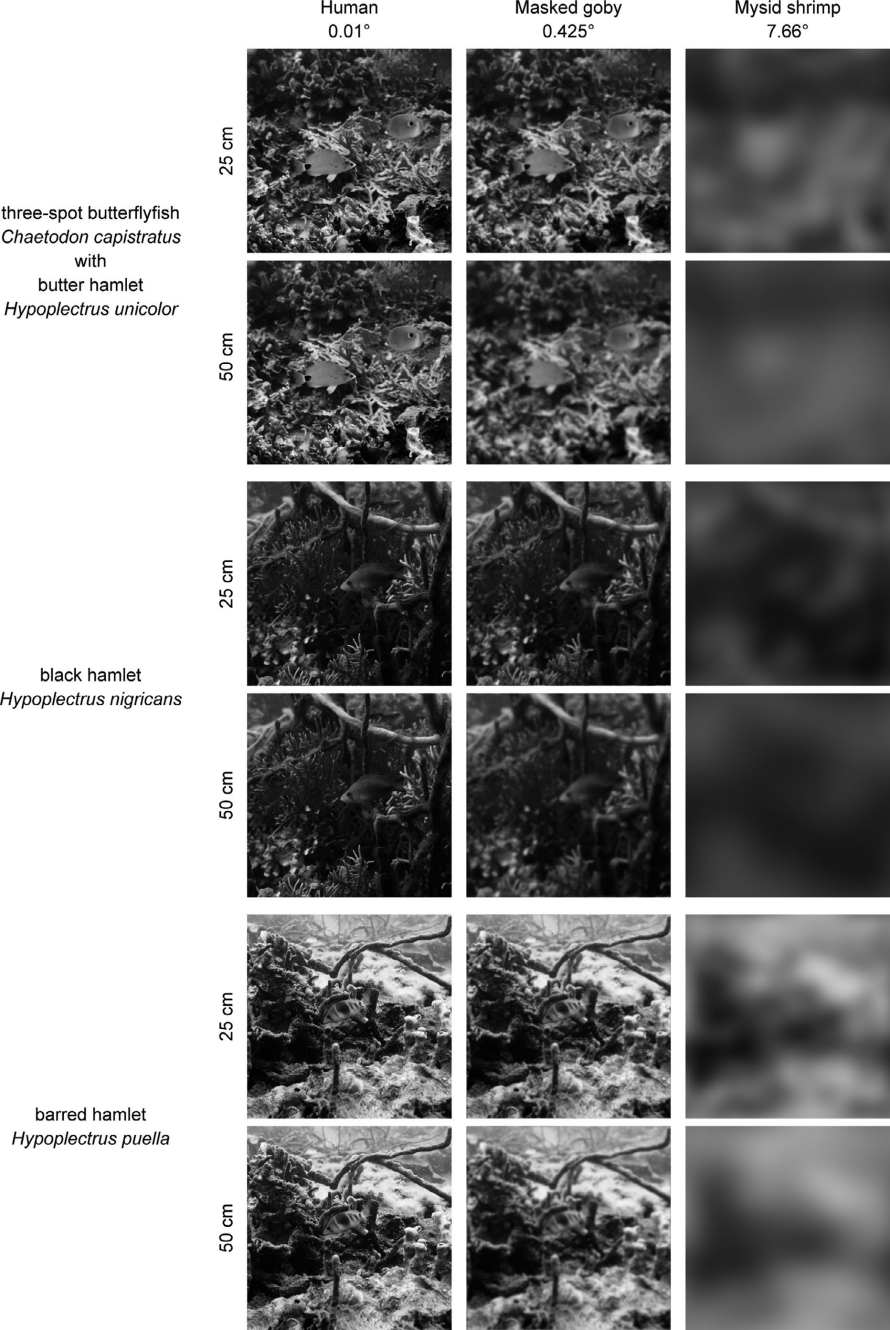
Monochromatic images of *Hypoplectrus* hamlets in a natural scene, at a depth of 5 m, on a coral reef in Bocas del Toro, Panama, as seen by the two main prey of hamlets, that is, the masked goby *C. personatus* and a *M. columbiae* shrimp, at the distances in which interactions between these species are known to occur. Acuity, expressed as minimum resolvable angle in degrees, is shown for each viewer

Modeling a mysid shrimp's visual system, which is devoid of color perception, revealed that only butter hamlets and their putative model butterflyfish could represent a valid model‐mimic pair, the difference in achromatic contrast between the two being well below the threshold of *Mysidium's* discrimination. On the contrary, our results suggest that the shrimp's visual system might have the potential to discriminate all other putative pairs on the basis of achromatic contrast. In conclusion, at least for the masked goby *C. personatus* and the mysid shrimp *M. columbiae*, known to be the principal diet items in the Bocas del Toro hamlet populations, the butter hamlet (*H. unicolor*) represents the only case consistent with an aggressive mimicry scenario, if color and/or luminance are used by the prey to discriminate friend (the model) from foe (the mimic). In addition, the limited visual resolution of both preys suggests that, at biologically significant distances, differences in the fine patterns over a relatively homogeneous yellow coloration of butter hamlet and butterflyfish are likely to be imperceptible. The co‐occurrence of model and mimic *H. unicolor‐C. capistratus* in Figure 6, as seen by a masked goby, provides hints on how the deceit might be obtained. The most relevant features shared by both model and mimic (Figure 6, second column, first and second row) are a uniform bright (yellow) body coloration with no vertical bars, a large black spot at the base of the tail highlighted by a white ring, the yellow tip of the snout, and the bright yellow pelvic fin.

An alternative strategy might have been taken by the black hamlet (*H. nigricans*). In the relatively turbid underwater conditions in Bocas del Toro, black hamlets can be quite hard to spot when dwelling between corals and overhangs, and this is even more the case for the generally limited spatial resolution of its prey. While it is unclear whether this dark cryptic coloration confers any advantage to the black hamlet in approaching its prey, it likely provides this morph some protection from its predators, as it does the dark brown cryptic coloration of its hypothetical model, the dusky damselfish *S. adustus*.

Unlike the non‐mimic barred hamlet *H. puella*, both model‐mimic pairs considered in this study are almost completely devoid of “patterns” consistent among individuals, with the exclusion of a large black eyespot at the base of the tail in both butterflyfish and hamlets and a fine UV pattern (invisible to the eyes of both masked goby and mysid shrimp) around the snout in the hamlets. A motive of thin black lines over yellow background in both butter hamlet and its model is visible to the human eye at close range and ideal illumination, but is much harder to detect in natural underwater illumination at any biologically meaningful distance. The anatomical acuity assays for both prey species and their representation in the Fourier image analysis suggest that at best only the black eyespot is discernible by either prey at the typical distances (25–50 cm) at which the species interact during a predation event, making color pattern analysis redundant for the purpose of this study focusing on these two pairs of models and mimics.

**FIGURE A4:**
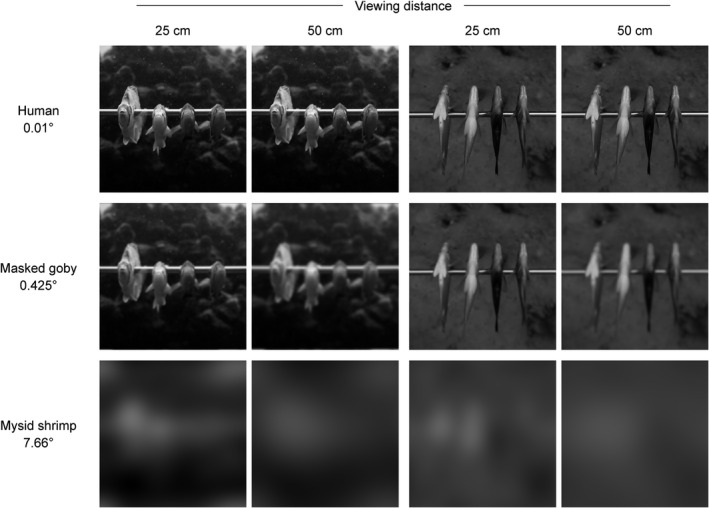
From left to right, in each image: Four‐eye butterflyfish (*C. capistratus*), butter hamlet (*H. unicolor*), black hamlet (*H. nigricans*) and barred hamlet (*H. puella*), seen 45° from below (*first two columns, left*). Detail of ventral appearance (*last two columns, right*)

The non‐mimic barred hamlet (*H. puella*) sports complex color patterns with high‐contrast dark vertical bars and considerable differences in luminance and hue with both model species. A barred hamlet at close distance from its prey is likely to be easily identified by the typical highly contrasting dark vertical bars over a comparatively brighter yellowish body, even with the very limited acuity of a goby, as suggested by the Fourier transform analysis (Figure 6). It is interesting to note that barred and more complex disruptive patterns are also typical of other sympatric basses and all invariably attack their prey almost horizontally while close to the substrate. On the contrary, butter hamlets are often seen attacking from about 45 degrees above masked gobies (*pers. obs*.). From this line of view the hamlet silhouette and color is remarkably similar to that of a four‐eye butterflyfish, in particular the very conspicuous structural yellow pelvic fins (Figure A4). Notably, while the color of pelvic fins in hamlets is highly variable between individuals within morphs, from colorless to bright yellow to highly saturated indigo or blue, this does not seem to be the case for butter hamlets, invariably sporting bright structural yellow on their pelvics with very similar hue to that of four‐eye butterflyfish pelvics, as evidenced by the reflectance measurements and below threshold perceptual distances. Therefore, the observation that butter hamlets attack masked gobies from slightly above suggests they might be behaviorally optimizing the efficiency of their aggressive mimicry by presenting to the prey with best visual abilities, the masked goby, the closest model‐resembling area of their body. We did not observe such top‐down attack strategy by the butter hamlet when preying on mysids, possibly because of the mysid's limited visual system.

In conclusion, this set of results, together with previous behavioral work by Puebla et al. (2007, 2018) and Picq et al. (2019), lends support to the aggressive mimicry scenario for the butter hamlet and its model, the four‐eye butterflyfish, at least in the Bocas del Toro population, where the “model following” behavior was observed and where this study was conducted. However, our results suggest that the main prey of butter hamlets, epibenthic masked gobies and mysid shrimp, might have not been the intended signal receiver driving the evolution of mimetic coloration, given that its main prey species are either completely devoid or possess very limited color vision. On the contrary, predators of hamlets, such as groupers and snappers, are generally at least fully dichromatic when not trichromatic, and represent more likely receivers of *H. unicolor's* mimic coloration than its own prey. This raises the possibility that the color patterns of the butter hamlet might not have evolved first for efficient aggressive mimicry by imitating appearance and behavior of a fish harmless to their prey but that other selective forces initially brought about the similar appearance which was later recruited for aggressive mimicry. Picq et al. (2019) found that the model‐tracking aggressive mimicry behavior of butter hamlets represents in fact one of two alternative behavioral syndromes, associated with territoriality. Territory holders with defined permanent hide‐outs only rarely engaged in aggressive mimicry, while roaming individuals, lacking a defined permanent hide‐out and territory systematically took advantage of this behavioral strategy. The authors proposed that the aggressive mimic strategy might confer an advantage in terms of foraging opportunities, at the expense of higher exposure to predators and possibly fewer mating opportunities. In this context, before aggressive mimicry evolved, it would have been beneficial for exposed roaming individuals to mix with and mimic, in behavior and appearance, a coral reef fish species with simple color patterns and unpalatable for a wide number of small‐to‐medium sized hamlet predators. Adult butterflyfish in the Caribbean are not a common occurrence in stomach content records (Randall, 1967), a testament to their effective defenses, mainly high maneuverability, extremely deep bodies with long, robust spines, particularly in benthivore species, a challenge for their gape‐limited predators (Hodge et al., 2018). In addition, *C. capistratus* sports less complex color patterns than other Caribbean butterflyfish, a potentially easier starting point for the development of a hamlet mimic. The protection conferred to roaming hamlets by mimicking in behavior (Picq et al., 2019; Puebla et al., 2007) and appearance a less than desirable prey in the eyes of their potential predators, such as di‐ or trichromatic groupers and snappers with sharp acuity (Loew & Lythgoe, 1978; Losey et al., 2003; McComb et al., 2013), could have led to the butter hamlet mimic morph. On the contrary, the limited visual system of the most common hamlet prey, masked gobies and *Mysidium* shrimp, represent an unlikely source of selection for the origination (but not the maintenance) of the mimic butter hamlet. Although the close resemblance between butter hamlet and four‐eye butterflyfish is unlikely to have evolved to deceive the visual system of the prey, and therefore not in the context of aggressive mimicry, the resemblance resulting from selection for protective mimicry did eventually start giving butter hamlets an advantage (Puebla et al., 2007) in accessing their prey. While not exerting direct selection on the butter hamlet's color patterns, aggressive mimicry is likely a significant contributor to the maintenance of this behavioral strategy.

Our study shows how the study of sensory systems not only broadens our understanding of animal communication and signaling but has the potential to generate new hypotheses on the origin and maintenance of mimicry and the evolutionary trajectory of species.

## CONFLICTS OF INTEREST

None declared.

## AUTHOR CONTRIBUTION

**Michele E. R. Pierotti:** Conceptualization (lead); Data curation (lead); Formal analysis (lead); Funding acquisition (lead); Investigation (lead); Methodology (lead); Project administration (lead); Software (equal); Supervision (lead); Validation (lead); Visualization (equal); Writing‐original draft (lead); Writing‐review & editing (lead). **Anna Wandycz:** Data curation (supporting); Formal analysis (equal); Methodology (supporting); Software (equal). **Pawel Wandycz:** Data curation (equal); Software (supporting); Visualization (supporting); Writing‐review & editing (supporting). **Anja Rebelein:** Investigation (supporting). **Vitor H. Corredor:** Investigation (supporting); Methodology (supporting); Writing‐original draft (supporting); Writing‐review & editing (supporting). **Juliana H. Tashiro:** Investigation (supporting); Methodology (supporting); Writing‐original draft (supporting); Writing‐review & editing (supporting). **Armando Castillo:** Investigation (supporting); Visualization (supporting); Writing‐review & editing (supporting). **William T. Wcislo:** Funding acquisition (supporting); Project administration (supporting); Resources (supporting); Writing‐review & editing (supporting). **Owen McMillan:** Funding acquisition (supporting); Project administration (supporting); Resources (supporting); Writing‐review & editing (supporting). **Ellis Loew:** Conceptualization (equal); Formal analysis (supporting); Methodology (supporting); Writing‐review & editing (supporting).

## Data Availability

Pierotti et al. Aggressive mimicry in hamlet fish: Complete results of PERMANOVAS and bootstrap analyses of dS, dL, are archived into the public repository Dryad https://doi.org/10.5061/dryad.jwstqjq6f

## Appendix 1 Additional methods and results

### Underwater irradiances

We used an Ocean Optics USB2000 spectrometer connected to an Ocean Optics ZPK600 UV/VIS optical fiber and fitted at one end with a CC‐3‐UV cosine corrector. The probe was secured at the tip of the longer arm of a white 1 m long L‐shaped pole and directed by a scuba diver vertically upwards and downwards, and horizontally perpendicularly to the shoreline, toward and away from shore, and the two directions parallel to the shoreline. This was repeated at different depths (just below surface, 2.5 m, 5.0 m, 7.5 m) just above hamlet territories. All measurements were taken between 11.00 and 13.00 on a sunny clear day.

The spectral distribution of irradiance was characterized by calculating *λ*P_50_, the wavelength that halves the total number of photons (Munz & McFarland, 1973), and the breadth of the light spectrum by calculating the difference Δ*λ* between *λ*P_25_ and *λ*P_75_ cumulative photon frequency, in the range 350–650 nm, most relevant for visual functions. The depth and wavelength dependence of the downwelling irradiance can be expressed in terms of the diffuse attenuation coefficient, *K*_d_(*λ*) (Mobley, 1994):

$$\frac{E_{d}\left(z,\lambda \right)}{E_{d}\left(0,\lambda \right)}=\exp\left[‐\int_{0}^{z}K_{d}\left(z^{\prime }\lambda \right)dz^{\prime }\right]$$

where $E_{d}(z,\lambda )$ is the downwelling irradiance at depth *z* and $E_{d}(0,\lambda )$ is the downwelling irradiance just below the water surface. If we consider the average ${\bar{K}}_{d}\left(z,\lambda \right)$ over the depth interval from below the surface to the maximum depth recorded, this is

$${\bar{K}}_{d}\left(z,\lambda \right)=‐\frac{1}{z}\ln\frac{E_{d}(z,\lambda )}{E_{d}(0,\lambda )}$$

We calculated the average diffuse attenuation coefficient using the below surface and 7.5 m depth downwelling spectral irradiance measurements.

#### Results

Peak irradiance of downwelling light measured just under the surface was reached at about 505 nm (1.531 × 10^14^ photons cm^–2^ s^–1^ nm^–1^) and the corresponding total downwelling irradiance in the interval 350 nm–650 nm was 2.47 × 10^16^ photons cm^–2^ s^–1^ nm^–1^. Peak sidewelling irradiance just under the surface was reached at the same wavelengths (3.46 × 10^13^ photons cm^–2^ s^–1^ nm^–1^). The total sidewelling irradiance just under the surface was 5.36 × 10^15^ photons cm^–2^ s^–1^ nm^–1^. The contribution of upwelling irradiance at the same depth was predictably more limited: the maximum upwelling irradiance was reached at about 508 nm (7.77 × 10^12^ photons cm^–2^ s^–1^ nm^–1^) and the total upwelling irradiance under the surface was 1.24 × 10^15^ photons cm^–2^ s^–1^ nm^–1^.

The downwelling irradiance had, in general, similar characteristics to those measured in other tropical reefs (McFarland & Munz, 1975) but with a more pronounced effect of attenuation, particularly at longer wavelengths. The average diffuse attenuation coefficient of the downwelling irradiance was highest at 680 nm (*K_d_* = 0.143/m) with a second maximum in the near‐UV (395 nm, *K_d_* = 0.106/m). The minimum was attained at 350 nm, the shortest wavelength recorded (*K_d_* = 0.031 m) and a second minimum at 530 nm (*K_d_* = 0.044 m) (Figure A1). This pattern of higher attenuation with depth at short and long wavelengths is mirrored by the narrowing of spectral bandwidth (Δ*λ*) with depth (Figure 1, main text; Figure A1). In clear atoll waters, at 5 m depth, McFarland and Munz (1975) reported a Δ*λ* = 105 nm, while at Punta Caracol, in Bocas del Toro, at a similar depth (5.0 m) we measured a substantially narrower bandwidth (Δ*λ* = 75 nm), a value observed only at 20 m in their study.

### Microspectrophotometry methods

Fish were maintained under dark conditions for a minimum of four hours prior to MSP and then euthanized with an overdose of MS‐222 followed by cervical dislocation. The eyes were rapidly enucleated under dim red light, and the retinas removed and maintained in PBS (pH 7.2) with 6% sucrose. Small pieces of the retina were placed on a cover slide, fragmented to isolate individual photoreceptors, and sealed with a second cover slide and Corning High Vacuum grease. We used a single‐beam, computer‐controlled MSP, with a 100 W quartz iodine lamp that allowed for accurate absorption measurements down to 340 nm (Loew, 1982; Losey et al., 2003). The peak of maximum absorption (*λ*_max_) of photoreceptors was obtained by fitting A1 or A2 templates to the smoothed, normalized absorbance spectra (Govardovskii et al., 2000; Lipetz & Cronin, 1988). We used the criteria for data inclusion into the analysis of *λ*max described in Loew (1994) and Losey et al. (2003).

**TABLE A1:** Distribution of cone visual pigments in the gobiid species examined to date

Species	ROD	SWS	MWS	LWS	Habitat	References
*Gobiusculus flavescens*	508	453	531	557	Temperate reef	Utne‐Palm and Bowmaker (2006)
*Pomatoschistus minutus*	508	447	527	548	Temperate reef	Jokela‐Määttä et al. (2009)
*Gobius paganellus*	512	465	565	565	Temperate reef	Loew and Lythgoe (1978)
*Asterropteryx semipunctata*	498	–	531	538	Tropical coral reef	Losey et al. (2003)
*Coryphopterus personatus*	501	–	531	539	Tropical coral reef	This study

### Visual modeling

Photoreceptor quantum catch *Q_i_* for a receptor of class *i* was calculated as:

$$Q_{i}=\int_{\lambda }R_{i}\left(\lambda \right)S^{a}\left(\lambda \right)I\left(\lambda \right)d\lambda$$

where $R_{i}\left(\lambda \right)$ is the absolute spectral sensitivity for receptor class *i*, *S^a^*(*λ*) is the reflectance spectrum of a color patch *a*, *I*(*λ*) is the irradiance spectrum, and integration is over the range *λ* = 350÷650 nm. Given the small distances (*d* = 25–50 cm) over which models and mimics interact with the receiver (i.e., the prey: masked goby or mysid shrimp) in clear coral reef waters, we considered light attenuation effects negligible.

If we take the signal *f_i_* of each receptor of class *i* as proportional to the natural logarithm of the receptor quantum catch *q_i_*, according to Weber‐Fechner's law, the contrast between two homologous patches, for example, on the model versus on its mimic, will be:

$$\Delta f_{i}=\;\ln\left[q_{i}\left({\text{patch}}_{1}\right)\right]‐\ln\left[q_{i}\left({\text{patch}}_{2}\right)\right]$$

The Vorobyev‐Osorio model assumes that discrimination thresholds are limited by photoreceptor noise. Color contrasts Δ*S* between corresponding patches on model and mimic viewed by the goby were calculated as:

$$\Delta S^{2}=\frac{{\left(\Delta f_{2}‐\Delta f_{1}\right)}^{2}}{e_{1}^{2}+e_{2}^{2}}$$

for a dichromatic species with short‐wavelength (1) and long‐wavelength (2) sensitive photoreceptors, with *e*_1_, *e*_2_, representing the photoreceptor noise associated with each class. For a trichromatic species with short‐ (1), medium‐ (2) and long‐wavelength (3) sensitive photoreceptors, the expression becomes:

$$\Delta S^{2}=\frac{e_{1}^{2}{\left(\Delta f_{3}‐\Delta f_{2}\right)}^{2}+e_{2}^{2}{\left(\Delta f_{3}‐\Delta f_{1}\right)}^{2}+e_{3}^{2}{\left(\Delta f_{2}‐\Delta f_{1}\right)}^{2}}{{\left(e_{1}e_{2}\right)}^{2}+{\left(e_{1}e_{3}\right)}^{2}+{\left(e_{2}e_{3}\right)}^{2}}$$

In relatively bright light conditions, as in the case of shallow waters in well‐illuminated coral reefs, the photon shot component of noise is negligible and neural noise will be largely accounting for the photoreceptor noise *e_i_*. Neural noise is inversely proportional to the relative frequency of the receptor types as given by the following equation:

$$e_{i}\approx \omega /\sqrt{\eta_{i}}$$

where *ω* is the Weber fraction and *η_i_* the relative density of photoreceptors of type *i*. For the masked goby, we set a Weber fraction *ω* = 0.05 for the long wavelength sensitive cone, a conservative estimate for fishes (Champ et al., 2016; Cheney & Marshall, 2009). During preliminary MSP work, we did not find any evidence of short‐wavelength sensitive cones in this species. Taking a conservative approach, we decided to consider both a dichromat scenario with the MSP values obtained in this study as well as a hypothetical trichromat condition, adding a blue‐sensitive cone located in a region of spectrum typical of other gobies (Table A1). For the dichromat, we set the relative proportions of the different cone types as 1:1 and at 1:4:4 for the trichromat scenario, to account for the apparent rarity (if present) of the putative short‐wavelength sensitive cone class in this species. The Weber fraction for the mysid was set at 0.05.

Masked gobies and mysids might be able to discriminate mimic hamlets from models based on differences in luminance. Based on studies of other fish and terrestrial animals (Kelber et al., 2003; Neumeyer et al., 1991), we assumed that the longwave sensitive photoreceptor in the masked goby and mysid shrimp is responsible for the achromatic perception of luminance. Thus, we computed the luminance contrast Δ*L* between patches on model and mimic as:

$$\Delta L=\Delta f_{3}/\omega_{3}$$

where *ω* = 0.05, a value close to that observed in other teleosts (Olsson et al., 2017). Color and luminance distances were calculated separately at three different depths, using horizontal irradiance (averaged over the 4 cardinal directions), measured at 2.5 m, 5.0 m and 7.5 m of depth, spanning the range of hamlet territories on the studied reef.

### Masked goby acuity

Optical properties of the eye and spacing of photoreceptors and retinal ganglion cells are considered the main anatomical factors limiting acuity, with the former generally playing a minor role in affecting visual resolution in fishes. While photoreceptor densities are intuitively expected to influence the minimum resolvable angle (Northmore & Dvorak, 1979), visual processing by neural cells in the retina and, in particular, visual summation by ganglion cells, can significantly reduce resolution (in favor of increased sensitivity). Higher‐level processing might, in certain cases, lead to further loss of spatial information (Warrant, 1999). This is consistent with the frequent observation of higher anatomical than behavioral acuity values in fishes.

The eyes of masked gobies were enucleated and fixed in paraformaldehyde (PFA) 4% diluted in phosphate‐buffered saline (PBS) for 3 hr, and then transferred to PBS and stored at 4ºC. The cornea and lens were removed and the retinas were dissected from the sclera. The free‐floating retinas were immersed in 10% hydrogen peroxide solution diluted in PBS, for 72 hr, at room temperature, for retinal epithelium bleaching. The retinas were washed in PBS and flattened onto a gelatinized glass slides with the ganglion cell layer facing up. The slides were exposed to 4% PFA vapors overnight, at room temperature, in order to increase the adhesion of the retina to the slide and for differentiation of the stained neurons (Coimbra et al., 2006; Stone, 1981). For Nissl staining, the tissues were rehydrated by passing through ethanol series in decreasing concentrations (95%, 70% and 50%) and distilled water acidified with glacial acetic acid. The retinas were stained in an aqueous solution of 0.1% cresyl violet at room temperature for approximately 3 min and dehydrated by passing through a series of ethanol and xylene. The slides were cover slipped using DPX (Sigma‐Aldrich, St. Louis, MO, USA). To evaluate shrinkage during the dehydration process, the areas of the retinas were measured before and after staining, using the software ImageJ (NIH, Bethesda, USA). Photographs of the retinas were taken with a digital camera (Axio CamMR, Carl ZeissVision GmbH, Germany), coupled to a stereomicroscope (SMZ775‐T, NIKON, Japan), and software (Axio Vision 4.1, Carl Zeiss, Germany).

To estimate the total population of GCL cells, we applied the optical fractionator method (West et al., 1991) with modifications for retinal wholemounts (Coimbra et al., 2009, 2012). The retina was considered a single section, and so the section sampling fraction was equal to one. The total population of cells $N_{\text{tot}}$ was estimated based on the total number of counted cells ΣQ and the area of the sampling fraction asf, which corresponds to the ratio between the counting frame and the sampling grid (Coimbra et al., 2009):

$$N_{\text{tot}}=\Sigma Q\frac{1}{\text{asf}}$$

The cells were counted using a Leica DM5500B trinocular microscope with a motorized stage, connected to a computer running the Stereo Investigator software (MicroBrightField, Colchester, VT). The edges of the retina and the optic nerve were delineated using a 5×/NA 0.15 objective. Counts were made using ×100/NA 1.4–0.7 oil immersion objective, at regular intervals defined by a sampling grid at 150 × 150 μm placed in a random, uniform and systematic fashion, covering the entire retinal area. An unbiased counting frame at 50 × 50 μm was imposed at each sampling frame and cells were counted if they lay entirely within the counting frame or if they touched the acceptance lines without touching the rejection lines (Gundersen, 1977). The coefficient of error was calculated using the method of Scheaffer et al. (1996). All cellular elements located within the GCL were counted, independent of size (Collin, 1989; Collin & Partridge, 1996; Collin & Pettigrew, 1988a, 1988b).

The theoretical spatial resolving power of each eye was estimated based on the maximum density of presumed retinal ganglion cells *D* and the focal length of the eye *f*, obtained from a Matthiessen's ratio of 2.34, appropriate for gobies (Hansen, 1988; Matthiessen, 1880; Ota et al., 1999; Wanzenbock et al., 1996). Considering a square array of retinal ganglion cells, the mean cell‐to‐cell spacing S is related to the maximum density *D* of ganglion cells per mm^2^ through the expression *S* ^2^ = 1/*D*. The maximum spatial (Nyquist) frequency (*ν*) of a sinusoidal grating resolvable by a square arrangement is *ν* = 1/(2ΔΦ), where ΔΦ is the inter‐receptor angle ΔΦ = *S*/*f* (Snyder & Miller, 1977). Substituting, we obtain:

$$\nu =1/\left(2\Delta \Phi \right)=f/\left(2S\right)\;\text{cycles}/\text{radian or}\nu =\;f/\left(2S\right)\;2\pi /360\;\text{cycles}/\text{degree},$$

or its inverse, the smallest resolvable angle *α*, in degrees.

### Image collection and processing

Underwater scenes were captured with either a Canon 5D Mark III camera with a 24–70 mm f2.8 lens set on 50 mm or with a Canon G7X, under natural illumination. Two sets of high‐resolution photographs were taken. First, hamlets and their models were photographed underwater at a depth of about 5 m, from an approximately horizontal line of view, against different natural backgrounds. In addition, during preliminary observations, we noted that the ventral silhouette of the butter hamlet *H. unicolor* closely resembles in shape and colors that of its putative model, the butterflyfish *C. capistratus*, despite having very different lateral profiles. In order to compare the appearance of the butter hamlet and its model (and, for comparison, the black hamlet *H. nigricans* and the barred hamlet *H. puella*), as seen from below at a 45° angle, as a masked goby hovering above a coral is likely to do, we collected one individual for each hamlet morph and a four‐eye butterflyfish. Fish were euthanized with an overdose of MS‐222 in an aerated aquarium then moved for few minutes at −20°C. A hole was then drilled on the dorsal area of each fish allowing a thin metal bar to be passed through the body so that the butter, black, and barred hamlets and the four‐eye butterflyfish could be placed side by side on a common base and brought underwater. A series of photographs were taken at a depth of 5 m in proximity to hamlets territories, at about 45° and 40 cm from the metal rod placed against different natural backgrounds. We did not note appreciable differences in color patterns between live non‐stressed individuals as observed in the wild and the sacrificed individuals as treated in our protocol, but their eyes did take a cloudy appearance and the tips of their fins showed some minor damage. However, it is significant that once we completed photographing and the bar was left for few minutes above a dead coral head, two hamlets, a blue and a butter, came immediately to inspect our setup and vigorously displayed against the "intruding" hamlets on the bar without interrupting their territorial displays even when we approached to try and recover the bar.

Following Caves and Johnsen (2018)’s guidelines, high‐resolution photographs were cropped to 1024 × 1024 pixels and saved separately for each RGB channel. The angular width of each scene was obtained by scaling with an object of known actual size in the photograph and by selecting a biologically significant viewing distance. For the photographs of live models and mimics in their natural habitat (Figure 6, main text), we measured the sizes of corals or rocks appearing in the images, while for the bottom‐up images of the mounted individuals (Figure A4) we measured the hamlets' inter‐orbital distances, to derive image width.
